# A Statistical Modeling of the Course of COVID-19 (SARS-CoV-2) Outbreak: A Comparative Analysis

**DOI:** 10.1177/1010539520928180

**Published:** 2020-05-25

**Authors:** Handan Ankarali, Seyit Ankarali, Hulya Caskurlu, Yasemin Cag, Ferhat Arslan, Hakan Erdem, Haluk Vahaboglu

**Affiliations:** 1 Istanbul Medeniyet University, School of Medicine, Department of Biostatistics and Medical Informatics, Istanbul, Turkey; 2 Istanbul Medeniyet University, School of Medicine, Department of Physiology, Istanbul, Turkey; 3 Istanbul Medeniyet University, School of Medicine, Department of Infectious Diseases and Clinical Microbiology, Istanbul, Turkey; 4 ID-IRI Lead Coordinator, Ankara, Turkey

**Keywords:** COVID-19, COVID-19 indicators, cubic model, nonlinear estimation, outbreak

## Abstract

This study aims to provide both a model by using cumulative cases and cumulative death toll for SARS-CoV-2 (severe acute respiratory syndrome coronavirus 2) outbreak in four countries, China, Italy, South Korea, and Turkey, starting from the first diagnosis and to compare associated indicators. The most successful estimation was obtained from the cubic model with natural logarithm for China, Italy, South Korea, and Turkey. The success of the models was around 99%. However, differences began to emerge in China, Italy, and South Korea after the second week. Although the highest number of new cases per 1 million people in China was 9.8 on February 28, 2020; it was 108.4 on March 21, 2020, in Italy; and this was 16.6 on March 5, 2020, in South Korea. On the other hand, the number of new cases was 24.6 per 1 million people on March 27, 2020, in Turkey. The log-cubic model proposed in this study has been set forth to obtain successful results for aforementioned countries, as well as to estimate the course of the COVID-19 outbreak. Other factors such as climacteric factors and genetic differences, which may have an impact on viral spreading and transmission, would also have strengthened the model prediction capacity.

## Introduction

The severe acute respiratory syndrome coronavirus 2 (SARS-CoV-2) outbreak has become the main concern for public health emergency particularly for the countries in the northern hemisphere.^1^ After starting in Chinese city of Wuhan on December 31, 2019, scientific research has focused on whether the disease will remain limited in a particular area with a small number of patients as were in SARS-CoV and MERS-CoV (Middle East respiratory syndrome coronavirus) outbreaks.

Artificial intelligence tools based on machine learning and mathematical models are being used to estimate the nature of the spread across each country, and to detect the potential amplifiers hampering the effects of the epidemic.^2^

This study aims to develop a model by means of the data from China, Italy, South Korea, and Turkey, by using cumulative cases and cumulative death toll starting from the diagnosis of the first case in each country.

## Materials and Methods

### Data Analysis

We used data of three countries (China, Italy, and South Korea) with a total number of cases exceeding 9000 as of March 26, 2020, as well as in Turkey within a 2-week period after the first case(s) of COVID-19 (coronavirus disease 2019).

#### China (Wuhan, Hubei)

The infection first emerged in Wuhan, the capital city of Hubei province, and then spread to the entire country during a very short time. However, the spread has been gradually brought under control through strict measures. The total number of cases till March 25, 2020, was 81 218. The main purpose of modelling of the outbreak in Hubei is to observe the natural course of the outbreak and its long-term consequences. Thus, insight for other countries, including Turkey, which is on the verge of the outbreak, can be obtained.

#### Italy

The first positive case was diagnosed on January 31, 2020. Data have been modeled for Italy because of the increasing number of cases and high mortality.^3^

#### South Korea

The first positive case was announced on January 20, 2020. Data have been modeled for South Korea because it is currently one of the countries that manages the outbreak very well.

#### Turkey

The first positive case was reported on March 10, 2020. Since the outbreak has just emerged in Turkey, data have also been modeled for Turkey as well.

### Indicators

Indicators utilized for modelling are the cumulative case and cumulative death toll for each day. For the comparison of countries, cumulative cases, cumulative death toll, and new cases were standardized by population size. The data were obtained from the World Health Organization and the Worldometer.^3-5^

Different nonlinear models were tested, and the results of the most suitable model are given.

### Model Validation for Suitability

For external validation, we examined whether the model types in each country are the same. In addition, the consistency between 16-day predictions obtained in this study and 10-day predictions in the study by Ankarali et al were accepted as internal validation.^6^

### Statistical Analysis

Exponential, quadratic, power, cubic, logistic, and growth curves were applied to the logarithmic values. In choosing the best predictive model, Akaike information criterion, prediction accuracy measure (R^2^ %), and mean squared error were used. Among the models, the most successful estimation was obtained from the cubic model. The appropriate mathematical model is as described below.

#### Cubic Model

The model that is defined as follows:

Ln(Y)=b0+(b1*t)+(b2*t**2)+(b3*t**3)

where *Y* are the cumulative case and cumulative death, *b* is the regression coefficient, and *t* is the time (day).

The criteria for performance and model coefficients of the selected model were given. GraphPad Prism (Trial Version 4.0a), Stata (Version 14), and SPSS (Version 23) programs were used.

## Results

**Figure 1.**
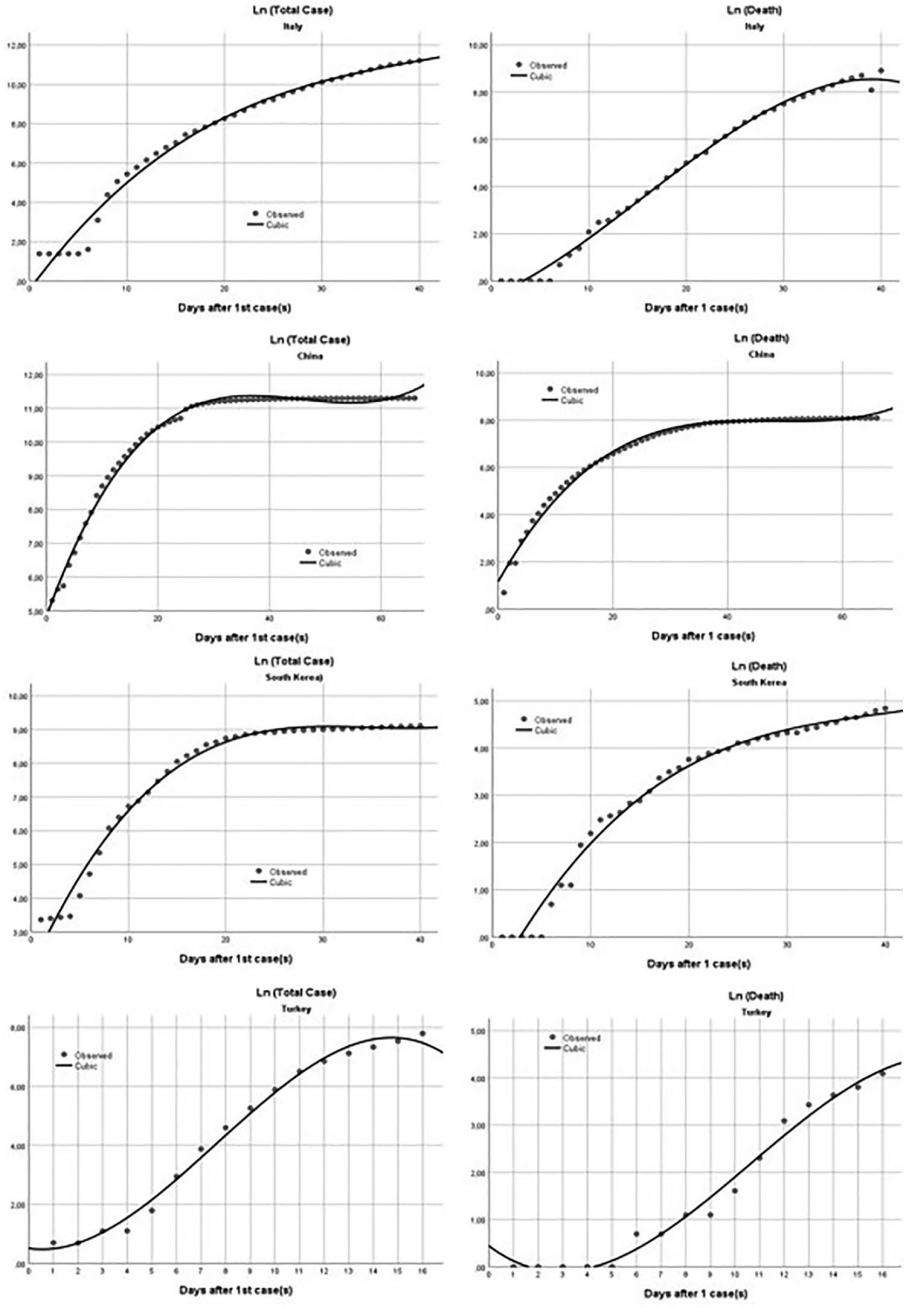
Observed versus predicted values according to the model in each country.

Table 1 and Figure 1 show the results of the model of the countries.

**Table 1.** Results of Prediction and Forecasting for Turkey.

Prediction date	Observed		Predicted					
	Total cases	Death	Total cases	95% CI for total cases		Death	95% CI for death	
March 25, 2020	2433	59	29 659	23 212	36 107	594	543	644
March 25, 2020	3629	75	39 381	32 666	46 096	747	695	800
March 27, 2020	5698	92	51 057	43 521	58 594	924	865	983
Forecast								
Forecast date	Total cases		95% CI for total cases		Death	95% CI for death		
March 28, 2020	64 866		55 661	74 071	1126	1054		1198
March 29, 2020	80 984		69 107	92 861	1354	1261		1447
March 30, 2020	99 587		83 993	115 181	1611	1489		1733

Abbreviation: CI, confidence interval for predictions/forecasting.

Determination coefficient of the models was calculated to be around 99%. When the results are examined, the outbreak is expected to end in the second week of April in China and in the beginning of April in South Korea. However, no significant decrease is observed in Italy. Since the evaluation period is too short, further prediction of the outbreak process cannot be made for Turkey.

When the four countries have been compared in terms of their total case prevalence, the results were similar for the first 15 days. However, differences have emerged after the second week in China, Italy, and South Korea. The number of cases per population was lowest in China, in the 22^nd^ day of the outbreak.

On the 22^nd^ day, the number of cases was lower in Italy than in South Korea. The breaking point in South Korea was around the 20^th^ day, and in China after the 25^th^ day. However, the breaking point for Italy and Turkey has not yet occurred.

The total death prevalence per 1 million people in China and South Korea for the first 15 days were similar. However, the course of the outbreak remained unclear in Turkey.

The number of new cases per 1 million people was lowest in China generally. In South Korea, the number of new cases observed was higher than in China, especially between the seventh and 20^th^ days. This prevalence was highest in Italy and had a very different course than the other three countries.

Although the highest number of new cases per 1 million people in China was 9.8 on February 28, 2020, it gradually decreased to 0.54 per 1 million people on March 25, 2020, which was similar to that of the new cases on January 20, 2020. Considering the date December 31, 2019, as the start of the outbreak, the duration to the culmination of new cases was 59 days (December 31, 2019, to February 28, 2020).

The highest number of new cases per 1 million people in Italy was 108.4 on March 21, 2020. However, it ended up with 79.2 per 1 million people on March 25, 2020. Considering the date February 15, 2020, as the start of the outbreak in Italy, the duration to the culmination of new cases was 37 days (February 15, 2020, to March 21, 2020).

While the highest number of new cases per 1 million people in South Korea was 16.6 on March 5, 2020, it ended up with 1.25 per 1 million people on March 24, 2020, which was similar to that of the cases on February 18 to 19, 2020. Considering February 15, 2020, as the start of the outbreak, the duration to the culmination of new cases was 15 days (February 15, 2020, to March 5, 2020).

Although the number of new cases increased day by day in Turkey, the rate started to decrease in the past 2 days. On March 27, 2020, 18 days after the first diagnosis appeared in Turkey, the number of new cases was 24.6 per 1 million people. Although this number was lower than total cases seen 18 days after the first diagnosis appeared in South Korea, it was higher than total cases seen 18 days after the first diagnosis appeared in China. China has reached this number in just one day and remained below in the remaining days.

## Conclusion

The prediction of the spread of the outbreak process is of great importance for future measures to be taken. In this study, we show that the cubic curve modeling can be successfully adapted to the four countries’ epidemic data, which include the total number of confirmed cases and death toll. China and South Korea, as the primary centers of the epidemic, provide significant input on how epidemic dynamics can be altered through preventative measures implied. In contrast, the experience in Italy, where the outbreak has still not been brought under control, will remain a significant observation for future studies. On the other hand, a breakpoint for Turkey, which is currently at the initial phase of the epidemic, could not be determined. However, since the selected model offers reliable results, the model coefficients will be re-estimated at 5-day intervals by adding the daily data and forecasting will be revised and recalculated for the next 5 days.
